# Nerve Identification Procedures Are Necessary for Complete Recovery From Recurrent Cases of Anterior Cutaneous Nerve Entrapment Syndrome: A Case Report

**DOI:** 10.7759/cureus.26497

**Published:** 2022-07-01

**Authors:** Tomoya Tsuchida, Yoshitaka Kondo, Kosuke Ishizuka, Takahide Matsuda, Yoshiyuki Ohira

**Affiliations:** 1 Department of Internal Medicine, St. Marianna University School of Medicine, Kanagawa, JPN; 2 Department of Gastroenterological Surgery, Okayama University Graduate School of Medicine, Okayama, JPN

**Keywords:** triggering nerve, lidocaine, abdominal pain, laparoscopic surgery, recurrence, acnes

## Abstract

Anterior cutaneous nerve entrapment syndrome (ACNES) involves pain in the abdominal wall due to nerve compression or ischemia. The diagnosis of ACNES is challenging with the pain often inclined to be diagnosed as psychological in origin. A 20-year-old woman presenting with abdominal pain was initially diagnosed with mesenteric lymphadenitis and prescribed pain relievers. However, following worsened pain, she was hospitalized. Blood examinations, abdominal and gynecological ultrasonography, and gastrocolonoscopy yielded no abnormal findings, leading to suspicions of psychological factors. As the patient experienced sharp abdominal pain on movement, but not at rest, which was temporarily relieved by lidocaine injections, she was diagnosed with ACNES. Rectus abdominal resection was performed but the pain relapsed. Laparoscopic surgery was performed to cut the nerve that caused the pain. After three surgeries, the patient was completely symptom-free. ACNES should be considered as a differential diagnosis for intractable abdominal pain. For recurrent relapses, the triggering nerves must be carefully identified for the successful treatment of ACNES.

## Introduction

Anterior cutaneous nerve entrapment syndrome (ACNES) is a type of abdominal wall pain caused by anterior cutaneous nerve compression or ischemia. Although local lidocaine injection is effective for both diagnosis and treatment, surgery may be required in some lidocaine-resistant cases [1]. However, if a diagnosis cannot be made, the pain is suspected to be of psychogenic origin. Therefore, it is necessary to establish alternative diagnostic and treatment strategies for cases that do not respond to lidocaine. We experienced a case of refractory ACNES wherein three surgeries were required for successful treatment. Herein, we present the events leading up to recovery and the treatment plan.

## Case presentation

Case history

A 20-year-old woman visited a general hospital with a slight fever and right lower abdominal pain. Blood tests showed no notable abnormalities while abdominal computed tomography revealed swollen lymph nodes around the ileocolic region. She was diagnosed with mesenteric lymphadenitis and was prescribed acetaminophen. Although the symptoms improved temporarily, the right lower abdominal pain worsened a month later. The patient was hospitalized owing to severe abdominal pain and referred to the Department of Gastroenterology at our hospital. Her body mass index was 19 kg/m^2^, and although she had a history of constitutional jaundice, she reported no habit of drinking or smoking. There was no family history of abdominal pain-related disease or resistance to lidocaine.

Differential diagnosis, investigations, and treatment

Physical examination at admission revealed localized tenderness in the epigastric region and lower right abdomen. Blood tests showed no notable abnormalities, and abdominal and gynecological ultrasonography did not show any abnormalities including those related to the mesenteric lymph node. There were no abnormal findings on gastrocolonoscopy. After admission, psychological factors were suspected. A psychiatrist examined the patient but did not identify any obvious mental illness.

The patient experienced brief but sharp abdominal pain on movement of the body but not at rest. The patient could not consume sufficient food at meals because of her abdominal pain while sitting. On the seventh day of hospitalization, we received a medical examination request to investigate the cause of abdominal pain. There were two localized tender points, both of which had an area < 2 cm^2^. One was at a right lower abdominal point and the other was 3 cm above the navel. The Carnett's sign was positive. Hypoesthesia was observed at the same site. We confirmed that there was no obvious lesion caused by the abdominal wall ultrasound in the painful area. We injected 5 ml of 1% lidocaine subcutaneously into the painful lesion and the pain improved soon after. Thus, based on all observations, we diagnosed the patient with ACNES. Two days later, the symptoms reoccurred, necessitating subcutaneous administration of the same amount of lidocaine at the same site. The patient was discharged; however, her symptoms subsequently relapsed. Although local injection of methylprednisolone 40 mg and lidocaine 5 mL was administered at the same site, her symptoms relapsed three days later. Moreover, the oral administration of pregabalin and loxoprofen was ineffective.

The patient then visited a pain specialty outpatient clinic and was prescribed duloxetine. However, she discontinued the medication because of nausea. She was no longer able to move because of abdominal pain. We concluded that the patient needed surgery; however, as our hospital has no experience with ACNES surgery, we referred her to a surgeon in another hospital.

**Figure 1:**
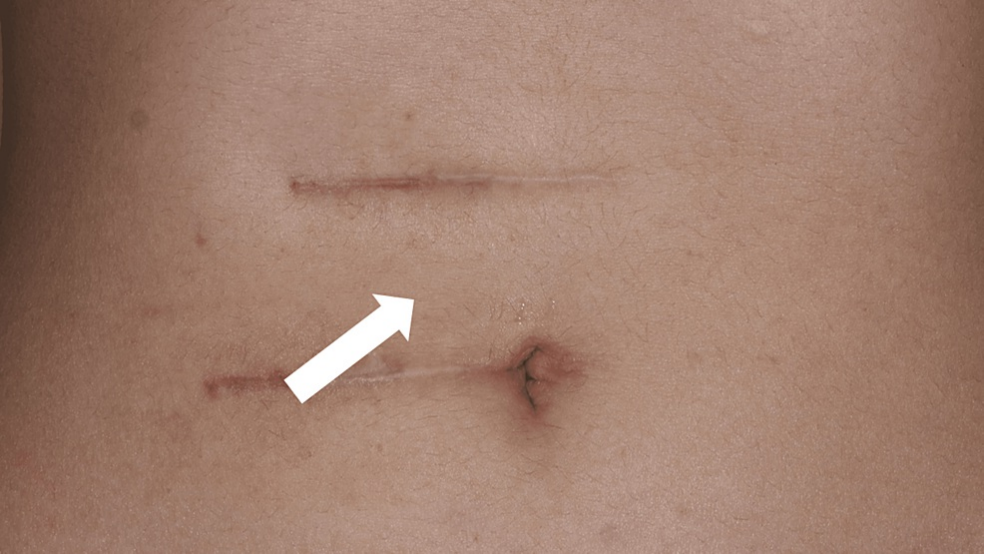
Patient's abdomen depicting painful area (white arrow) situated between her surgical scars

On March 25, X + 1, the patient underwent rectus abdominis muscle resection at the same site, which improved her symptoms. She was discharged from the hospital after rehabilitation. After surgical treatment, she reported no pain for some time. However, in July of the same year, after gastroenterocolitis, the pain recurred in two places outside the surgical scar. While local injection of lidocaine into the painful area relieved the pain for one day, the pain recurred shortly after. We concluded that ACNES had recurred. As she was unable to move, the patient could no longer lead a normal daily life. On January 27, X + 2, she revisited the same hospital and underwent a second rectus abdominis muscle resection. Although the mild pain improved for several days after the operation, the pain recurred in the areas between the wounds of the surgery during rehabilitation (Figure 1).

The patient was re-examined at our hospital after discharge. Her activities of daily living declined remarkably, and she could not go to the restroom without her mother’s help. As bathing or visiting the hospital became arduous for the patient, a home-visit bathing service and home-visit medical care, respectively, were initiated. A nerve block injection administered under echo guidance at another hospital was ineffective. The patient became mentally unstable. We referred her to a hospital for laparoscopic nerve dissection of ACNES. The patient was transferred to the hospital on October 6, X + 2. The improvement of tenderness was confirmed by local injection of lidocaine to the painful region. However, the patient complained of pain relapse at a slightly distant position the next day. A transverse abdominis plane block was performed, which was very effective in eliminating the pain. A continuous nerve block was started on the posterior sheath of the rectus abdominis muscle. After a week of continuous block, continuous analgesia and mental stability were achieved. The patient then started rehabilitation. As a result of rehabilitation under pain relief for a week, the patient was able to sit upright without significant pain.

Scar pain was likely to be exacerbated by simply removing the painful area. In addition, it was likely that wounds would cause iatrogenic ACNES because persistent pain had lowered the patient’s pain threshold. Therefore, we decided to perform a nerve incision at the surface of the transversus abdominis membrane through minimally invasive laparoscopic surgery using 3-mm (diameter) forceps.

Since the patient was in a continuous nerve block state on the posterior sheath of the rectus abdominis muscle, the pain of ACNES could be reproduced by low-frequency pulse stimulation on the transversus abdominis muscle membrane. The nerve responsible for her pain was identified. After marking the same site with Indocyanine Green, anesthesia was introduced and laparoscopic anterior cutaneous nerve ablation was performed on October 15, X + 2. Epidural anesthesia was terminated on October 19 and the ACNES pain disappeared completely. Both pre- and postoperative diagnoses were ACNES caused by the right nerve branch of Th10.

Outcome and follow-up

The patient was discharged on October 22 and was able to regain full quality of her daily life.

## Discussion

ACNES, a disease that causes abdominal wall pain, was first described by William V Applegate in 1972 [2]. This condition is considered to be the most common cause of abdominal wall pain [2,3]. It is estimated that 2% of emergency room patients with acute abdominal pain experience ACNES [4]. It has been hypothesized that pain will occur due to intra- or extra-abdominal pressure, local ischemia, and nerve compression caused by a hernia of the fat pad that protects the fibrous duct surrounding the nerve, resulting in strangulation [5]. Several factors can trigger ACNES onset, including trauma, surgical scars, rapid exercise, abdominal wall muscle weakness, obesity, ascites, and pregnancy [6]. Sudden changes in body weight may also be the cause. Occasionally, however, no clear factor is identified [7]. Abdominal wall perception consists of the anterior and lateral intercostal nerve branches of Th7-12. This nerve runs between the internal oblique and lateral abdominal muscles, reaching the skin through the neurovascular channel in the rectus abdominis muscle at the posterior sheath of the rectus abdominis muscle (at the lateral one-third of the rectus abdominis muscle). It is related to the perception of the abdominal wall [8].

In this case, ileocecal lymphadenitis and gastroenteritis may have been the trigger at the first and second visits. The second postoperative relapse is thought to have been caused by surgical scarring. The diagnostic criteria of ACNES (Table 1) were used [9].

**Table 1:** Diagnostic criteria for ACNES

Criteria	
1	Localized pain at the lateral border of the rectus abdominis muscle
2	Most intense pain at an area as large as one fingertip
3	Positive Carnett test
4	Positive skin pinch test and/or altered skin perception to light touch and/or cold temperature at the area of most intense pain;
	Normal diagnostics (blood/urine analysis and imaging); and
	≧50% pain reduction ±15 minutes after abdominal wall injection using a local anesthetic
	ACNES, anterior cutaneous nerve entrapment syndrome

Differential diagnoses include abdominal wall hernia, multiple neuropathies with underlying diseases such as diabetes, spinal diseases such as cancer metastasis to the spine, and endometriosis [5]. Before lidocaine injection, it is necessary to confirm that there is no obvious abnormality in the abdominal wall by performing ultrasonography or computed tomography.

Boelens et al. reported the treatment of 139 patients with ACNES [1], 81% felt 50% relief after the first injection, and 33% felt permanent relief after only one injection. Fifty percent (n = 69) patients had neurectomy, of whom 71% (n = 49) were successfully treated. Chrona et al. created a treatment algorithm for ACNES, according to which in situations when the symptoms recur after lidocaine injection, the injection should be repeated. Additionally, if possible, an ultrasound-guided rectus abdominis sheath block, transverse abdominis muscle block, or steroid injection should be administered. If there is no further improvement, local injections, such as botulinum toxin preparations and alcohol, could be considered. If the pain is still untreatable, surgery must be implemented [10], as it was found to be effective in randomized controlled trials [11].

In our case, pain recurrence was observed even after several injections of lidocaine and steroids. Since we had no experience with treatment with muscle block under echo guidance, we decided to transfer the patient to another hospital for the operation. For cases with post-surgical relapse, additional surgery may be effective [12]. In this regard, the second rectus abdominis muscle resection was selected even after pain recurrence. However, relapse occurred twice. The pain was finally treated successfully at a facility with experience in laparoscopic surgery [13].

In this case, the problem was whether the pain could be relieved after nerve re-dissection at the same site. According to a previous paper, re-dissection at the same site could lead to improvement in some cases [12]. However, the second operation showed almost no effect in this case. As an actual treatment strategy, it is necessary to consider the pharmacological nerve block as an important judgment factor in order to determine the nerve dissection site appropriately before surgery.

Method 1

If the block to the nerve hole of the rectus abdominis muscle anterior sheath is functional, the nerve re-dissection in the rectus abdominis muscle anterior sheath will be effective. In our case, the nerve was cut twice, and the anterior sheath block was ineffective.

Method 2

The rectus abdominis muscle posterior sheath block had some effect. However, in the two operations, the invasion extended to the posterior sheath of the rectus abdominis muscle. We had concerns about the enlargement of the incision area. Nerve dissection on the posterior sheath might have prolonged postoperative wound pain.

Method 3

Transverse abdominis plane block was very effective (improved to NRS0-1). We decided to use laparoscopy for nerve dissection to reduce the surgical wound. We selected laparoscopic nerve dissection on the transversus abdominis membrane surface. The treatment was finally successful. Even in the case of re-dissection, it is necessary to divide the diagnostic block from the body surface to a deep location several times over several days. The location of the nerve dissection should be changed to the one where the nerve block works. Therefore, laparoscopic surgery is not always possible, even during the third resection as in our case, and the aforementioned methods 1 and 2 must be considered.

It is necessary to select the excision site and optimal treatment method after considering the level at which the nerve block is most effective, the level at which the nerve was cut in the previous surgery, and the extent of the scar. There are few reports of effective surgical treatments after ACNES double relapse. If the effect of the nerve block is confirmed and dissection performed at the same level, remission by surgical treatment can be expected as in our case, even after multiple nerve dissections.

ACNES is a treatable condition; however, it is highly likely to be overlooked. Patients with delayed diagnosis experience a great deal of anxiety. A psychiatric diagnosis (anxiety, physical symptoms, or depression) could be made. Antidepressants and tranquilizers may be prescribed [3]. If the pain relapses repeatedly, as in this case, the patient will be more likely to be perceived as having mental health issues. When a patient reports localized abdominal pain during movement in cases where no clear cause can be identified upon imaging examinations, we should list ACNES as a differential diagnosis and initiate the diagnostic treatment by lidocaine injection.

Even if relapse occurs, the pain disappears after lidocaine injections at the same site. Therefore, it is critical for clinicians to recognize the diagnostic bias of psychological factors. Moreover, it is necessary to determine the site at which nerve dissection would be most effective and recommend that the patient undergoes surgical treatment at a hospital with experience in treating such cases.

## Conclusions

We experienced a case of recurrent ACNES that required three surgeries for successful treatment. Physicians should consider ACNES as part of the differential diagnosis for patients who present with intractable abdominal pain. If a local injection is only temporarily effective, the next intervention step should include surgery. Even in cases of multiple relapses, it is necessary to identify the site of nerve dissection carefully. Subsequently, surgical treatment by a surgeon with experience in nerve dissection could be considered.
